# NFAT5 promotes arteriogenesis via MCP‐1‐dependent monocyte recruitment

**DOI:** 10.1111/jcmm.14904

**Published:** 2019-12-28

**Authors:** Xing‐Chi Lin, Miao Pan, Ling‐Ping Zhu, Quan Sun, Zheng‐Shi Zhou, Chuan‐Chang Li, Guo‐Gang Zhang

**Affiliations:** ^1^ Department of Cardiovascular Medicine Xiangya Hospital Central South University Changsha China; ^2^ Department of Laboratory Animal Xiangya School of Medicine Central South University Changsha China; ^3^ Department of Geriatric Medicine Xiangya Hospital Central South University Changsha China; ^4^ National Clinical Research Center for Geriatric Disorders Xiangya Hospital Central South University Changsha China; ^5^ Department of Cardiovascular Medicine The Third Xiangya Hospital Central South University Changsha China

**Keywords:** arteriogenesis, hindlimb ischaemia, macrophage, monocyte chemoattractant protein 1, nuclear factor of activated T cells 5

## Abstract

Studies have demonstrated that nuclear factor of activated T cells 5 (NFAT5) is not only a tonicity‐responsive transcription factor but also activated by other stimuli, so we aim to investigate whether NFAT5 participates in collateral arteries formation in rats. We performed femoral artery ligature (FAL) in rats for hindlimb ischaemia model and found that NFAT5 was up‐regulated in rat adductors with FAL compared with sham group. Knockdown of NFAT5 with locally injection of adenovirus‐mediated NFAT5‐shRNA in rats significantly inhibited hindlimb blood perfusion recovery and arteriogenesis. Moreover, NFAT5 knockdown decreased macrophages infiltration and monocyte chemotactic protein‐1 (MCP‐1) expression in rats adductors. In vitro, with interleukin‐1β (IL‐1β) stimulation and loss‐of‐function studies, we demonstrated that NFAT5 knockdown inhibits MCP‐1 expression in endothelial cells and chemotaxis of THP‐1 cells regulated by ERK1/2 pathway. More importantly, exogenous MCP‐1 delivery could recover hindlimb blood perfusion, promote arteriogenesis and macrophages infiltration in rats after FAL, which were depressed by NFAT5 knockdown. Besides, NFAT5 knockdown also inhibited angiogenesis in gastrocnemius muscles in rats. Our results indicate that NFAT5 is a critical regulator of arteriogenesis and angiogenesis via MCP‐1‐dependent monocyte recruitment, suggesting that NFAT5 may represent an alternative therapeutic target for ischaemic diseases.

## 1 INTRODUCTION

Although interventional therapy and bypass surgery are effective treatments for patients with coronary heart disease (CHD), these procedures are not suitable for some patients and are associated with a high postoperative restenosis rate.^1^, ^2^ Well‐developed coronary collateral circulation (CCC) is crucial for improving the symptoms and prognosis of CHD patients.^3^, ^4^ Therefore, investigating the regulatory mechanism of CCC formation and promoting the opening and development of CCC to reconstruct a patient's autologous blood supply may be a promising solution to those patients.

Arteriogenesis, the basic process of CCC formation, during which pre‐existing small arterioles are remodelled into large and functional collateral arteries to divert blood flow around stenotic lesions, is a complex set of events involving interactions among various cell types and signalling circuits.^5^, ^6^ Inflammation plays a crucial role in arteriogenesis.^7^ Shear force can induce activation of endothelial cells (ECs), with a subsequent up‐regulation of cell adhesion molecules such as intercellular adhesion molecule‐1 (ICAM‐1), vascular cell adhesion molecule‐1 (VCAM‐1) and chemokines such as monocyte chemoattractant protein 1 (MCP‐1), these factors further recruit monocytes which secrete matrix metalloproteinases (MMPs) and growth factors, leading to rearrangement of the extracellular matrix and outward remodelling of the arterioles.^8^, ^9^, ^10^, ^11^ However, the intracellular modules regulating MCP‐1 release and monocytes recruitment during arteriogenesis are still unclearly.

The transcription factor, nuclear factor of activated T cells 5 (NFAT5), belongs to the Rel family, which includes the NF‐κB and NFATc proteins.^12^, ^13^ Originally described as a hypertonicity‐responsive transcription factor that orchestrates cellular homeostasis, NFAT5 has also been implicated in regulating the expression of genes associated with migration and proliferation of cells.^14^, ^15^ NFAT5 mediates the migration and proliferation of smooth muscle cells, and thus promotes arterial stiffening.^16^, ^17^ Besides, NFAT also involves in vascular endothelial growth factor (VEGF)‐mediated human umbilical vein endothelial cell (HUVECs) migration and angiogenesis.^18^ More importantly, arterial wall stress, which plays critical part in arteriogenesis, activates NFAT5 in cultured vascular smooth muscle cells (VSMCs) which may contribute to an improved migratory activity of VSMCs and thus promote maladaptive vascular remodelling processes.^19^

NFAT5/MCP‐1 axis has been suggested to play considerable roles in inflammation‐related diseases. NFAT5 regulates macrophage apoptosis, survival and proliferation by inducing MCP‐1 secretion and thereby plays a central role in the pathogenesis of macrophage‐dependent chronic inflammatory diseases such as rheumatoid arthritis.^15^ Besides, in human kidney, the high interstitial sodium concentration in the medulla generates a defence zone with enhanced antibacterial immunity via NFAT5/MCP‐1‐dependent monocytes chemotaxis, mononuclear phagocytes bactericidal activity and cytokine production.^20^ However, it is not clear whether MCP‐1 release and monocytes recruitment during arteriogenesis are determined by NFAT5.

In this study, we will demonstrate that NFAT5 controls MCP‐1 release in ECs, as well as promotes monocytes recruitment and arteriogenesis in hindlimb ischaemia after femoral artery ligation (FAL), in order to discover a new mechanism and provide a novel therapeutic target for the development of collateral circulation.

## 2 MATERIALS AND METHODS

### 2.1 Rat hindlimb ischaemia model

All animals care protocols and experiments were reviewed and approved by the Animal Care and Use Committee of the Department of Laboratory Animals, Xiangya School of Medicine, Central South University. All of the rats were maintained in the specific pathogen‐free facility of the Department of Laboratory Animals, Central South University. 250‐300 g male Sprague‐Dawley (SD) rats were anaesthetized with 3% pentobarbital sodium (50 mg/kg) by intraperitoneal injection, and the surgical procedures were performed under sterile conditions. For hindlimb ischaemia model, we performed femoral artery ligature (FAL) referencing previous studies.^21^, ^22^ Briefly, a vertical longitudinal incision was made in the left hindlimb, and the femoral artery and its branches were then dissected and ligated. For the arteriogenesis experiments, adductor muscles were harvested 3 or 21 days after surgery. For the angiogenesis experiments, gastrocnemius muscles were harvested 7 or 21 days after surgery. The muscles were fixed in 4% paraformaldehyde (PFA) for further experiments. For MCP‐1 supplement experiments, MCP‐1 (450 ng/mL, Abcam) in phosphate‐buffered saline (PBS) or PBS alone was infused via osmotic minipumps (10 μL per hour for 7 days, DURECT Corporation) after surgery, as described previously.^23^

### 2.2 Postmortem angiography

For postmortem angiography, lead sulphate/gelatin contrast agent was infused into rat hindlimbs as previously described.^22^ X‐ray pictures were taken in a X‐ray chamber (model DHF‐155H, HITACHI MEDICAL CORPORATION) exposed to 50 kV and 100 mA for 30 ms Pictures were taken and transferred to a computer where identification and counting of collateral arteries were performed. Angiographies were obtained after 21 days of the FAL.

### 2.3 NFAT5 shRNA adenovirus production and administration

The adenoviral vectors carried rat NFAT5 shRNA (Ad‐shNFAT5) or the negative control (Ad‐null) were constructed by Genechem Co., Ltd. The shRNA sequences were as follows: Ad‐shNFAT5 (5′‐CCGGGCAATGGAAGTGTTGACTTGGCTCGAGCCAAGTCAACACTTCCATTGCTTTTTG ‐3′) and Ad‐null (5′‐CCGGTTCTCCGAACGTGTCACGTCTCGAGACGTGACACGTTCGGAGAATTTTTG‐3′). For NFAT5 knockdown experiments in rats, Ad‐shNFAT5 or Ad‐null was delivered (1 × 10^10^ PFU per rat) after being divided among four to five injection sites in the adductor or gastrocnemius and surrounding muscles 3 days before FAL.

### 2.4 Laser speckle imaging

Blood flow recovery was measured by scanning both rear paws with laser speckle contrast imaging (LSCI) (PeriCam PSI Z, Perimed) before and after the surgical procedure (0, 3, 7, 14 and 21 days). During the procedure, the animals were kept under pentobarbital sodium anaesthesia and their body temperatures were strictly maintained between 36.5°C and 37.5°C. Plantar perfusion was quantified within anatomically defined regions of interest (ROIs). The results were reported as the ratio of blood flow in the left to the right (L/R) hindlimb.^24^

### 2.5 Cell culture and transfection

Human umbilical vein endothelial cells were purchased from ScienCell Research Laboratories, Inc and maintained in endothelial cell medium (ScienCell) supplemented with 5% foetal bovine serum (FBS), endothelial cell growth supplement and 1% penicillin/streptomycin (ScienCell). THP‐1 cells were obtained from the Cell Bank of Type Culture Collection of Chinese Academy of Sciences and cultured in RPMI‐1640 medium (Gibco) supplemented with 10% FBS (ScienCell) and 1% penicillin/streptomycin solution. Both HUVEC and THP‐1 cells were maintained in a humidified atmosphere containing 5% CO_2_ at 37°C. The human NFAT5 siRNA (si‐NFAT5) and siRNA negative control (si‐NC) (Santa Cruz Biotechnology, Inc) were transfected to HUVECs for 24 hours using Lipofectamine 2000 (Life Technologies) according to the manufacturer's instructions.

### 2.6 Quantitative real‐time PCR

Total RNA was extracted from muscle or cell samples using RNAiso Plus reagent (TaKaRa), RNA concentration was quantitated by NanoDrop One (Thermo Fisher Scientific). For detection of NFAT5, ICAM‐1, VCAM‐1, MCP‐1 and GAPDH mRNA expressions, cDNAs were synthesized by using PrimeScript™ RT reagent kit with gDNA Eraser (TaKaRa) according to the manufacturer's instructions. Real‐time PCR was performed on the 7500 Real‐Time PCR System (Applied Biosystems) using SYBR Premix Ex Taq reagent (TaKaRa) with NFAT5, ICAM‐1, VCAM‐1, MCP‐1 and GAPDH specific primers. GAPDH was used as internal standard to normalize the mRNA expression level using 2^−ΔΔCt^ method. The sequences for each primer were shown in Table S1.

### 2.7 Western blot analysis

Muscle or cell samples were lysed for 30 minutes on ice in RIPA lysis buffer (Beyotime Institute of Biotechnology), and the protein concentration was determined by BCA Protein Assay Kit (Beyotime Institute of Biotechnology). Total protein (50 to 100 μg) was resolved by SDS‐polyacrylamide gel electrophoresis, transferred to a nitrocellulose membrane, and subjected to immunoblot analysis. The primary antibodies for NFAT5 (1:1000, Abcam), MCP‐1 (1:2000, Abcam), p‐ERK1/2 (1:2000, Cell Signaling Technology), ERK1/2 (1:1000, Cell Signaling Technology) or GAPDH (1:10 000, Abcam) and horseradish peroxidase‐conjugated secondary antibody (Santa Cruz Biotechnology) were used. The proteins were detected using enhanced chemiluminescence reagents (Millipore, Burlington), and bands were analysed with Image J software normalized by GAPDH.

### 2.8 Immunohistochemistry and immunofluorescence assay

We harvested the adductor or gastrocnemius muscles after FAL. The mid‐zone of the muscle (the 5‐mm‐wide centremost section) was trimmed. Samples were embedded in paraffin and 4 μm thick cross‐sections were performed for haematoxylin and eosin (HE) staining. For immunohistochemistry, we used antibodies to α‐SMA (1:800, Sigma), Ki‐67 (1:200, Abcam) and CD68 (1:200, Abcam). Paraffin section was rehydrated and endogenous peroxidase activity was blocked for 30 minutes in methanol which contains 0.3% hydrogen peroxide. Primary antibody was incubated at 4°C overnight, followed by 60 minutes for biotinylated secondary antibody (1:500, Abcam). All specimens were counterstained with haematoxylin staining solution (Beyotime Institute of Biotechnology), then neutral gum sealed piece for storage. Analysis of collaterals was performed after scanning the section through OLYMPUS CX41 and Leica Application Suite 4.0 software. For immunofluorescence assays, primary antibodies for NFAT5 (1:100, Abcam), MCP‐1 (1:100, Abcam), α‐SMA (1:400, Sigma) or CD31 (1:50, Abcam) were applied, secondary antibodies were used with labelling with Alexa Fluor dye (1:200, Abcam) with a maximum excitation at 488 nm (green), and for red with Alexa Fluor 594 nm. The slides were counter stained with DAPI (Sigma) to visualize cellular nuclei. Images were obtained by fluorescence microscope (DMI4000B, Leica) and analysed by Image‐Pro Plus 6.0.

### 2.9 ELISA assay

The concentration of MCP‐1 in the supernatant of the cultured HUVECs was determined by using enzyme‐linked immunosorbent assay (ELISA) kit (R&D Systems) according to the manufacturer's instructions.

### 2.10 Chemotaxis assay

4 × 10^5^ THP‐1 cells were placed in the upper chamber of a modified Boyden chamber (pore size 8 μm; Corning). The chambers were placed into 24‐well cell culture plate containing conditioned culture supernatant of the HUVECs treated with or without si‐NC, si‐NFAT5 or ERK1/2 inhibitor (U0126). After incubation for 3 hours at 37°C, the cells on the lower surface of the chamber were fixed with 4% PFA and counterstained with crystal violet and counted manually in five random fields using a microscope, the cells in the lower chamber were observed using a haemocytometer.

### 2.11 Statistical analysis

The data were presented as means ± standard deviation (SD). Differences between two groups were determined by Student's *t* test. Comparisons of multiple groups were performed with one‐way analysis of variance (ANOVA). All statistical analyses were performed using the SPSS 13.0 software package (SPSS Inc), and a two‐tailed *P* value < .05 was considered to be statistically significant.

## 3 RESULTS

### 3.1 NFAT5 is required for the development of collaterals in rats after hindlimb ischaemia

**Figure 1:**
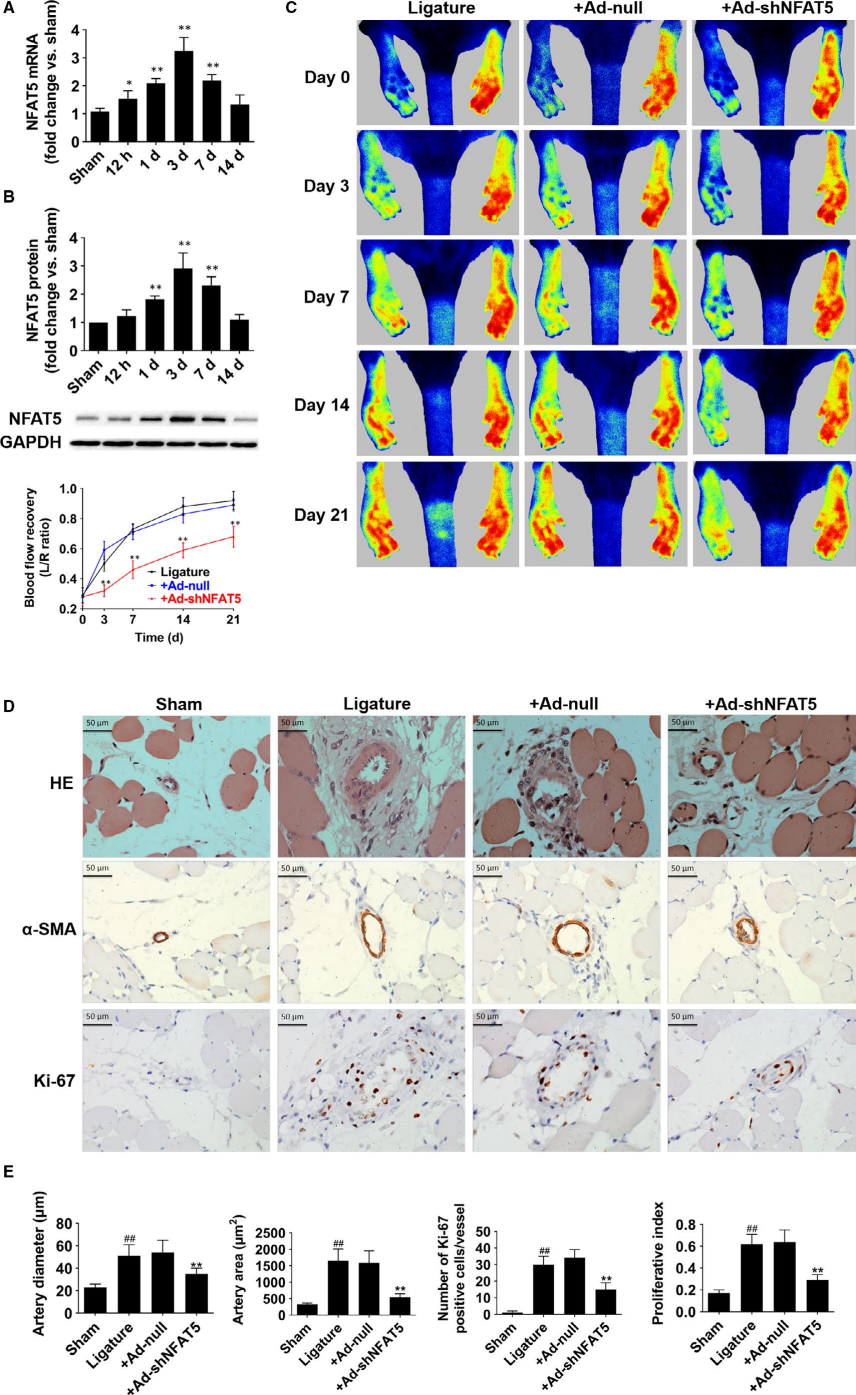
NFAT5 is required for collaterals formation in a rat hindlimb ischaemic model. A and B, NFAT5 mRNA and protein expression in adductor muscles at 12 h, day 1, day 3, day 7 and day 14 after FAL. C, Representative laser speckle perfusion images and statistical analysis of the ratios of left to right (L/R) hindlimb blood perfusion among rats treated with locally injection of Ad‐null and Ad‐shNFAT5 into adductor muscles immediately after (acute), 3 d after, 7 d after, 14 d after and 3 wk after FAL. D, HE staining, immunohistochemistry of α‐SMA and Ki‐67 in cross‐sections of the adductor muscles collected from sham‐, ligature‐, ligature + Ad‐null‐ and ligature + Ad‐shNFAT5‐treated rats 21 d after FAL. E, Quantification of images from (D), collateral artery diameter and lumen areas were assayed by α‐SMA staining, and proliferating cells were measured by Ki‐67 staining. *P < .05, **P < .01 for multi‐time points vs sham (A and B); **P < .01 vs + Ad‐null, ^##^ *P* < .01 vs sham (C and E); N = 6 rats in each group

In order to investigate whether NFAT5 participates in the development of collateral circulations, we performed hindlimb ischaemia in rats by FAL, which induced arteriogenesis from pre‐existing collateral anastomoses. Rats were subjected to FAL at one side and the other side was unligated hindlimb that was regarded as sham control. We detected NFAT5 expression in adductor muscles, found that both NFAT5 mRNA and protein expressions were increased at day 1, 3, 7 after FAL when compared with sham hindlimb and showed the most significant increase at day 3 (Figure 1A,B). Meanwhile, we also found the similar result that NFAT5 was activated by hypoxia in HUVECs (Figure S1A,B). We furtherly examined whether NFAT5 knockdown could reduce collateral circulations formation via locally injection of NFAT5 shRNA adenovirus into hindlimb muscles. As a result, NFAT5 mRNA and protein expressions in adductor muscles were increased at day 3 after FAL but both were remarkably decreased when treated with Ad‐shNFAT5 (Figure S2A,B). Meanwhile, double staining of NFAT5 and α‐SMA in adductor muscles 3 days after surgery indicated that NFAT5 was mainly located in ECs of collateral arteries and Ad‐shNFAT5 could markedly reduce NFAT5 expression in ECs of collateral arteries (Figure S2C). Besides, the number of angiographically visible collateral vessels was also significantly decreased in rats treated with Ad‐shNFAT5 compared with Ad‐null at day 21 after FAL (Figure S2D).

As arteriogenesis and collateral remodelling are the largest contributors to perfusion recovery after FAL,^25^ so we next investigated whether NFAT5 knockdown inhibits blood flow recovery using laser speckle contrast imaging at day 0, 3, 7, 14 and 21 after FAL. Consistent with the above, the Ad‐shNFAT5‐treated rats showed attenuated perfusion recovery in the hindlimbs as early as 3 days after surgery when compared with Ad‐null group (day 3, 32.3 ± 4.1% vs 59.1 ± 6.6%, *P* < .01; day 7, 46.5 ± 6.2% vs 71.8 ± 5.3%, *P* < .01; day 14, 59.5 ± 5.0% vs 83.2 ± 6.2%, *P* < .01; day 21, 68.3 ± 7.1% vs 89.2 ± 4.6%, *P* < .01; Figure 1C). Furtherly, HE staining and immunohistochemistry of α‐SMA (alpha‐smooth muscle actin) and Ki‐67 (a proliferation marker) in the adductors were used to determine the effects of NFAT5 knockdown on arteriogenesis (Figure 1D,E). At day 21 after FAL, the diameter of collaterals in Ad‐shNFAT5‐treated rats was smaller than those of control in Ad‐null‐treated rats (35 ± 5 vs 54 ± 11 μm, *P* < .01). Additionally, the artery area was also smaller in Ad‐shNFAT5‐treated group than those in Ad‐null‐treated (548 ± 102 vs 1584 ± 371 μm^2^, *P* < .01). To evaluate the proliferation of collateral arteries, we assayed cell proliferation in and around the collaterals by Ki‐67 staining. Ad‐shNFAT5‐treated rats showed lower Ki‐67 positive cells (15 ± 4 vs 34 ± 5, *P* < .01) as well as lower proliferation index (0.29 ± 0.05 vs 0.64 ± 0.11, *P* < .01) than those treated with Ad‐null. These results demonstrated that NFAT5 is required for collateral arteries growth which was inhibited by NFAT5 knockdown.

### 3.2 NFAT5 knockdown inhibits monocyte recruitment and MCP‐1 expression in rats hindlimb ischaemia model

**Figure 2:**
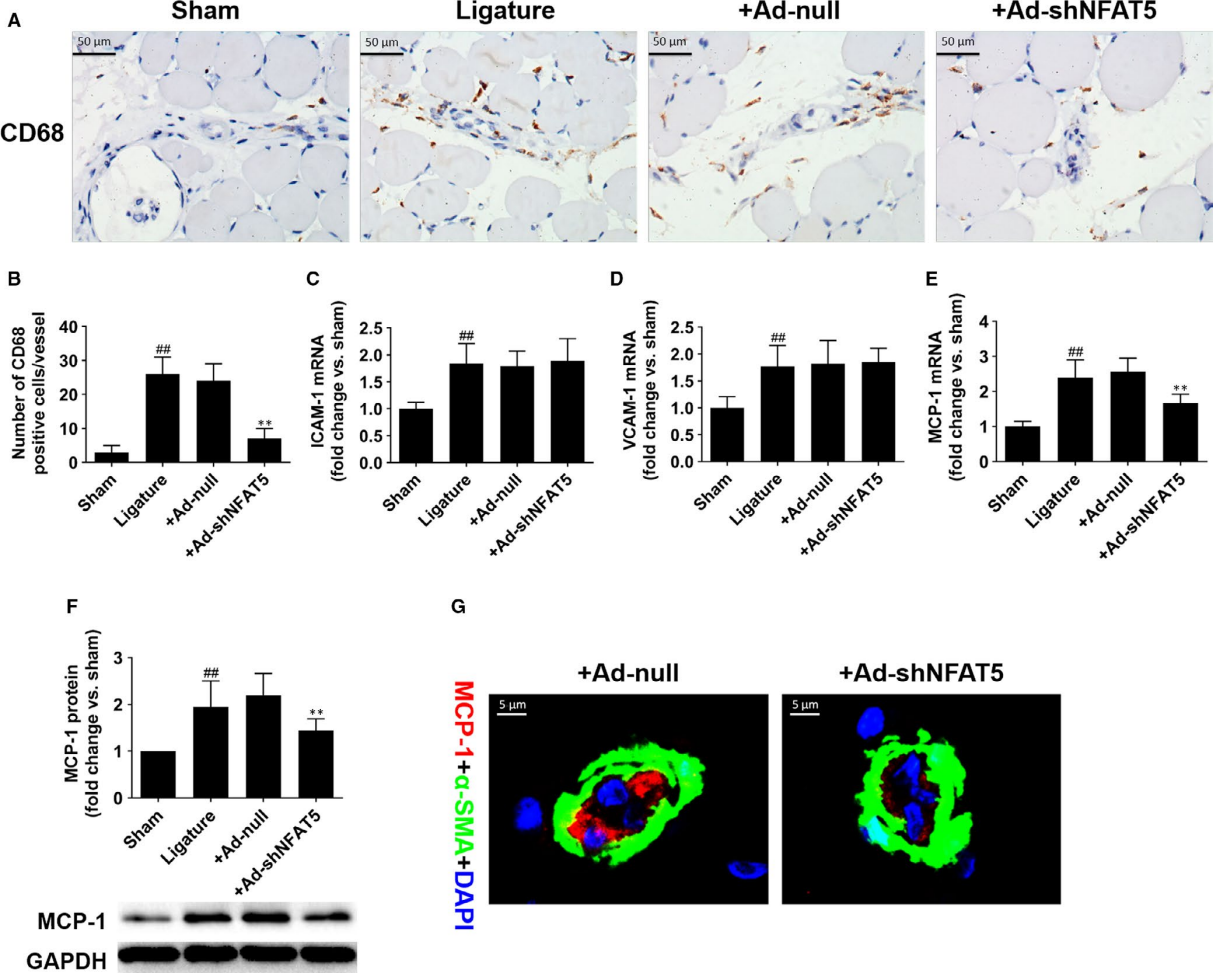
NFAT5 knockdown inhibits macrophages infiltration and MCP‐1 expression after hindlimb ischaemia surgery. A and B, Representative of immunohistochemistry staining and quantification of CD68 in adductor muscles selected from sham‐, ligature‐, +Ad‐null and + Ad‐shNFAT5 treated rats 3 d after FAL. C‐F, ICAM‐1, VCAM‐1 and MCP‐1 expressions in adductor muscles 3 d after indicated treatment. G, Representative immunofluorescence staining of MCP‐1 (red), α‐SMA (green) and DAPI (blue) in collateral artery 3 d after FAL. Scale bar = 50 μm or 5 μm. **P < .01 for vs + Ad‐null; ^##^ *P* < .01 vs sham; N = 6 rats in each group

Evidence suggested that inflammation is responsible for collateral vessel remodelling and NFAT5 is a critical regulator of inflammation,^7^, ^26^ so we therefore explored whether the infiltration of macrophages and secretion of inflammatory factors were affected by NFAT5 in vivo. Similarly, our results also demonstrated that collateral circulation growth was accompanied with increase of macrophage infiltration around the collateral vessels in the adductors, which was assessed by CD68‐positive staining (26 ± 5 vs 3 ± 2, *P* < .01, Figure 2A,B). Nevertheless, the infiltrating macrophages were significantly decreased when rats were treated with Ad‐shNFAT5 (7 ± 3 vs 24 ± 5, *P* < .01, Figure 2A,B). According to previous studies, ICAM‐1, VCAM‐1 and MCP‐1 are the three predominant molecules up‐regulated that induce monocyte recruitment during arteriogenesis.^8^, ^9^, ^10^, ^11^ So we investigated the expressions of these inflammatory factors in adductors 3 days after surgery and found that neither ICAM‐1 mRNA nor VCAM‐1 mRNA expression was affected by NFAT5 knockdown (Figure 2C,D). However, MCP‐1 mRNA and protein were significantly down‐regulated in rats treated with Ad‐shNFAT5 compared with Ad‐null (Figure 2E,F). In addition, double staining of MCP‐1 and α‐SMA in adductor muscles 3 days after surgery indicated that NFAT5 knockdown could markedly reduce MCP‐1 expression in the ECs of collaterals arteries (Figure 2G). These data suggested that NFAT5 knockdown results in the reduction of macrophage infiltration in adductors and MCP‐1 expression in ECs of collateral arteries.

### 3.3 IL‐1β promotes NFAT5 expression and translocation to the nucleus in HUVECs

**Figure 3:**
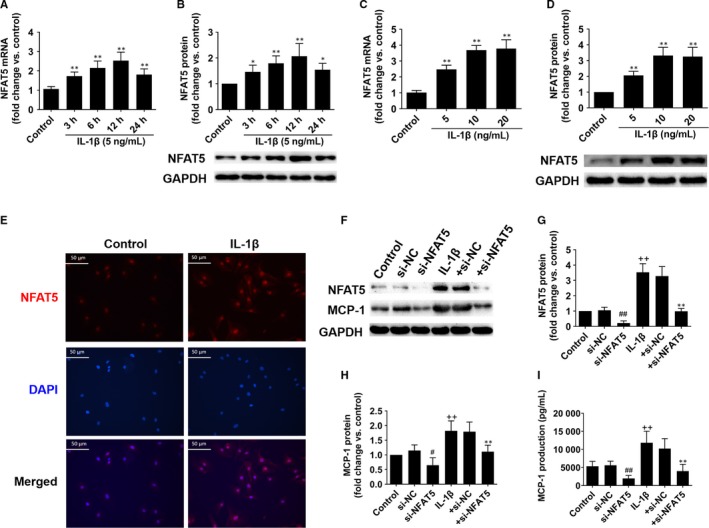
NFAT5 regulates MCP‐1 expression in HUVECs. A and B, NFAT5 mRNA and protein expressions in HUVECs treated with IL‐1β (5 ng/mL) at different time points. C and D, NFAT5 mRNA and protein expressions in HUVECs treated with different concentrations of IL‐1β for 12 h. E, Representative immunofluorescence staining of NFAT5 (red) and DAPI (blue) in HUVECs treated with IL‐1β (10 ng/mL) for 12 h. F‐H, Representative of Western blot images and quantification of NFAT5 and MCP‐1 protein expressions in HUVECs treated with si‐NFAT5 (20 nmol/L) or si‐NC (20 nmol/L) under basal or IL‐1β (10 ng/mL) stimulation for 12 h. I, The MCP‐1 concentration in culture supernatant of HUVECs treated with si‐NFAT5 (20 nmol/L) or si‐NC (20 nmol/L) under basal or IL‐1β (10 ng/mL) stimulation for 12 h. *P < .05, **P < .01, for IL‐1β vs control (A‐D), +si‐NC vs + si‐NFAT5 (G‐I); ^#^ *P* < .05, ^##^ *P* < .01 vs si‐NC (G‐I); ^++^ *P* < .01 vs control (G‐I). Each experiment was repeated three times. NC, negative control

As MCP‐1 released from ECs is an important mediator for arteriogenesis,^9^ which could be induced by interleukin‐1 β (IL‐1β),^27^ so we further investigated whether NFAT5 is involved in this process in HUVECs. Our results indicated that IL‐1β could apparently increase NFAT5 mRNA and protein expressions in time‐ and dose‐dependent manner in HUVECs (Figure 3A‐D), as well as promote NFAT5 translocation to the nucleus demonstrated by immunofluorescence assay (Figure 3E). Furthermore, we treated HUVECs with NFAT5 siRNA under both basal‐ and IL‐1β‐treated conditions, finding that NFAT5 and MCP‐1 protein expressions as well as MCP‐1 concentration in the culture supernatant were markedly increased stimulated by IL‐1β (Figure 3F‐I), but significantly decreased when treated with NFAT5 siRNA under both basal‐ and IL‐1β‐treated conditions (Figure 3F‐I).

### 3.4 NFAT5 enhances THP‐1 cell chemotaxis by regulating MCP‐1 release in HUVECs depending on activation of ERK1/2 pathway

**Figure 4:**
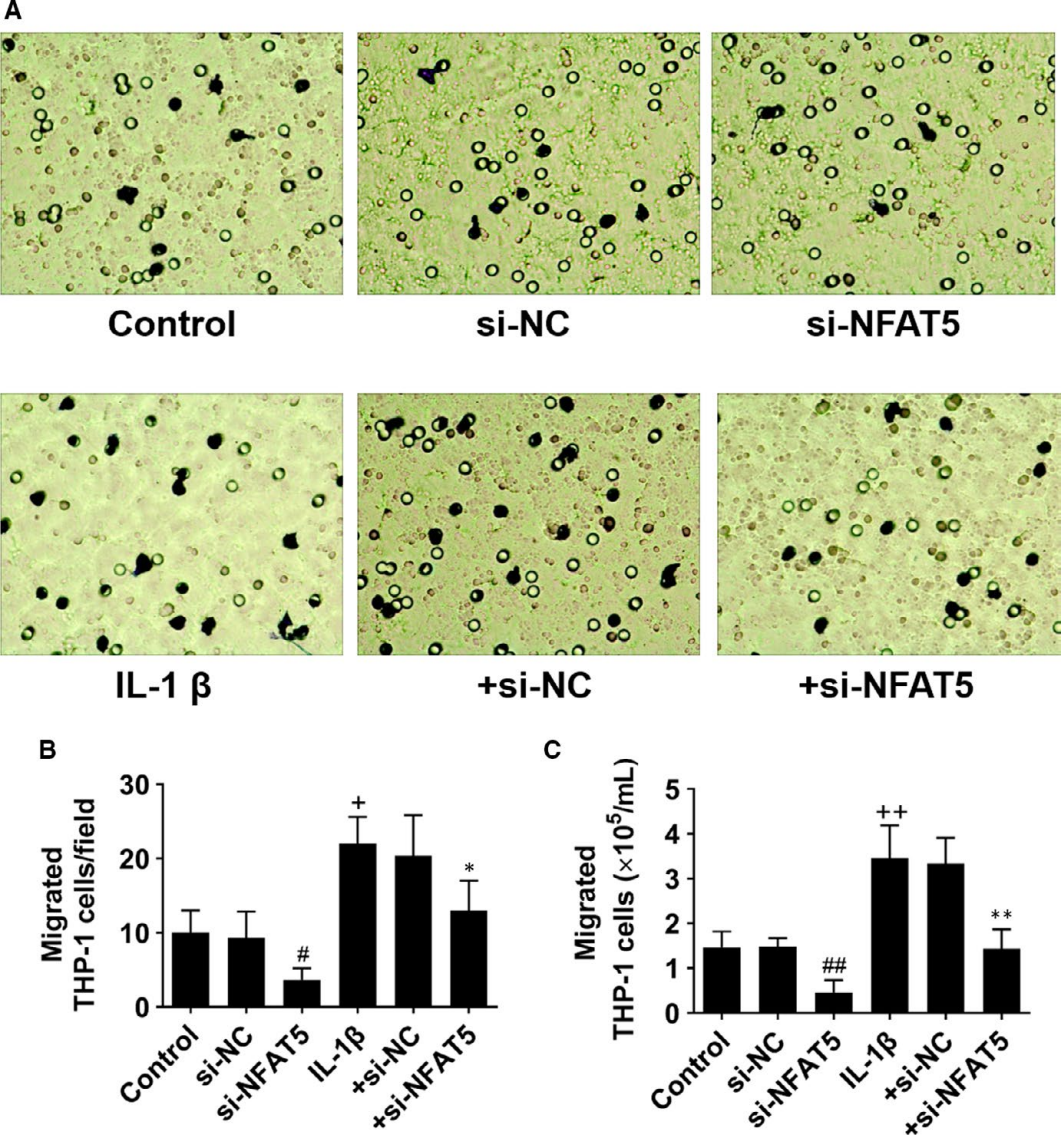
NFAT5 knockdown inhibits chemotaxis of THP‐1 cells. A, Representative images of migrated THP‐1 cells supplemented with the culture supernatant of HUVECs which had been incubated in si‐NFAT5 (20 nmol/L) or si‐NC (20 nmol/L) under basal or IL‐1β (10 ng/mL) stimulation for 12 h. B, The number of migrated THP‐1 cells on the reverse surface of the membrane. C, The number of migrated THP‐1 cells in the lower medium. *P < .05, **P < .01 vs + si‐NC; ^+^ *P* < .05, ^++^ *P* < .01 vs control; ^#^ *P* < .05, ^##^ *P* < .01 vs si‐NC. Each experiment was repeated three times. NC, negative control

As the chemotaxis and adhesion of monocytes to ECs are an important process of arteriogenesis,^9^, ^11^ so we next investigated whether NFAT5 in HUVECs affects the chemotaxis of THP‐1 cells in vitro using transwell migration assay to imitate the process during arteriogenesis in vivo. We firstly cultured HUVECs under different conditions and then investigated the chemotaxis of THP‐1 cells cultured in the culture supernatant of HUVECs. Our results indicated that IL‐1β could significantly induce chemotaxis of THP‐1 cells, but NFAT5 knockdown by NFAT5 siRNA markedly inhibited the migration of THP‐1 cells migrated into the lower surface of the chambers and the medium under both basal‐ and IL‐1β‐treated conditions (Figure 4A‐C). These data and the results suggested that NFAT5 is essential for IL‐1β‐induced THP‐1 cell chemotaxis.

**Figure 5:**
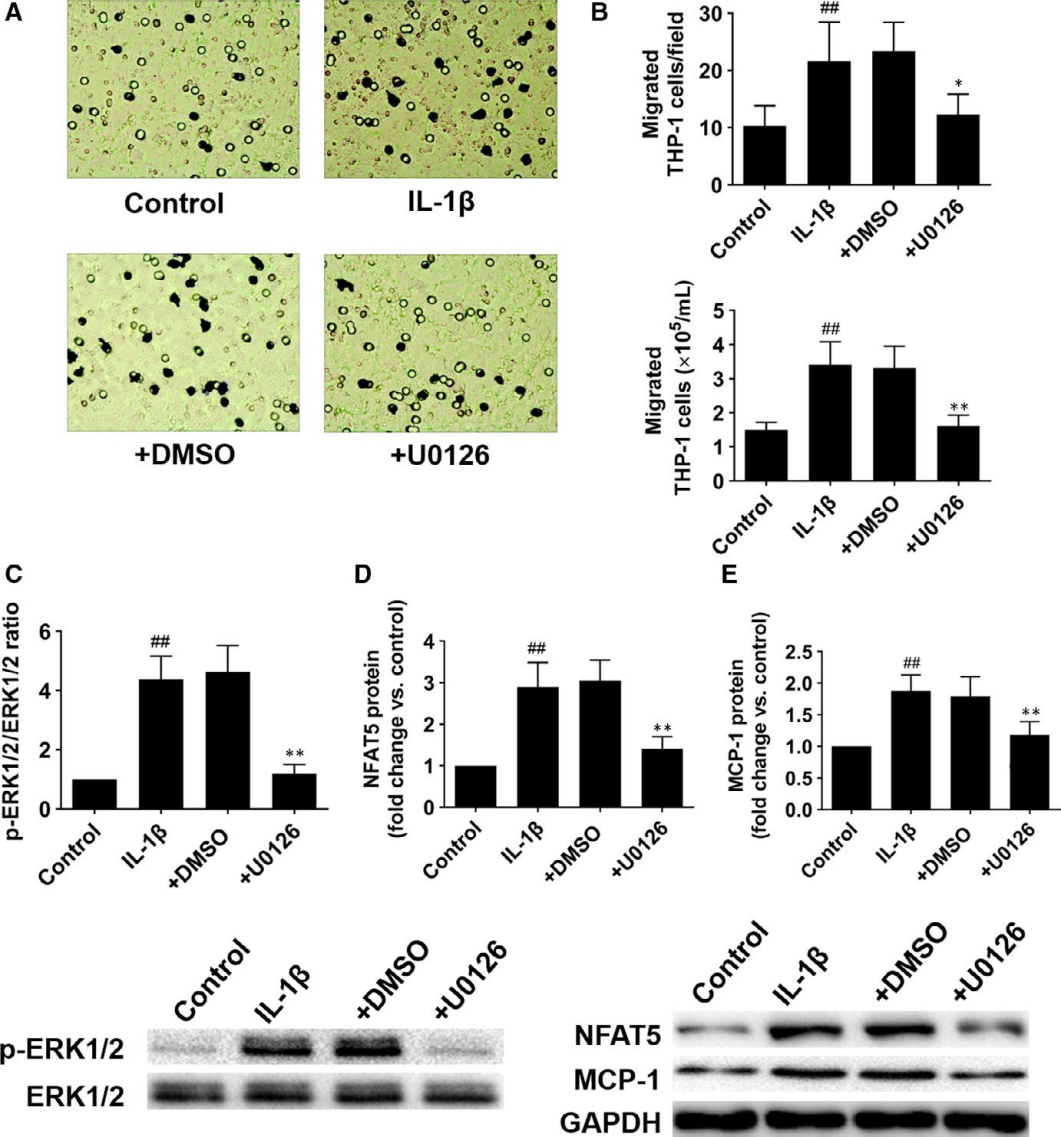
The ERK1/2 pathway involves in NFAT5‐mediated chemotaxis of THP‐1 cells. A, Representative images of migrated THP‐1 cells supplemented with the culture supernatant of HUVECs which had been pretreated with ERK1/2 inhibitor (U0126, 10 μmol/L) or DMSO for 30 min before treatment with IL‐1β (10 ng/mL) for an additional 12 h. B, The number of migrated THP‐1 cells on the reverse side of the membrane and in the lower medium. C‐E, ERK1/2, NFAT5 and MCP‐1 protein expressions in HUVECs treated as (A). *P < .05, **P < .01 vs + DMSO; ^##^ *P* < .01 vs control. Each experiment was repeated three times

Because activation of ERK1/2 pathway is required for IL‐1β‐induced MCP‐1 expression,^28^ so we further investigated the role of ERK1/2 pathway in NFAT5‐regulated chemotaxis of THP‐1 cells. Our results showed that ERK1/2 pathway inhibitor (U0126) could significantly inhibit IL‐1β‐induced THP‐1 cells migration (Figure 5A,B). Meanwhile, IL‐1β induced phosphorylation of ERK1/2 as well as increased NFAT5 and MCP‐1 protein expressions, which can be abolished by ERK1/2 inhibitor (Figure 5C‐E). All these data stated clearly that NFAT5 induced chemotaxis of THP‐1 cells through MCP‐1 which was depended on the activation of ERK1/2 pathway.

### 3.5 MCP‐1 delivery reverses the inhibitory effects of NFAT5 knockdown on collateral circulations growth in rats

**Figure 6:**
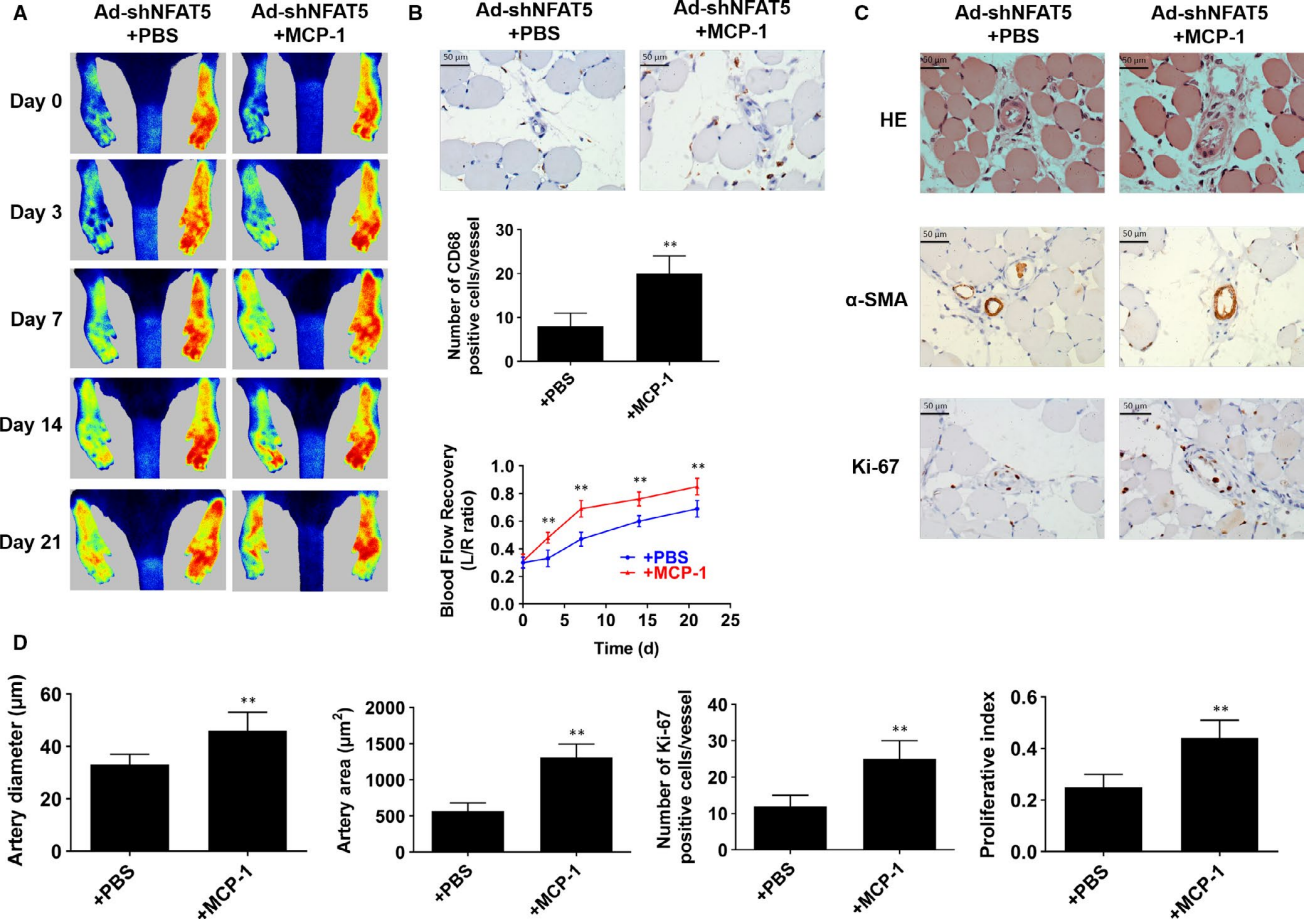
MCP‐1 delivery recovery arteriogenesis which is inhibited by NFAT5 knockdown. A, Representative images of laser speckle perfusion images and statistical analysis of the ratios of left to right (L/R) hindlimb blood perfusion in NFAT5 knockdown rats treated with MCP‐1 delivery at various time points after FAL. B, Representative of immunohistochemistry staining and quantification of CD68 in adductor muscles selected from NFAT5 knockdown rats treated with MCP‐1 delivery 3 d after FAL. C, HE staining, immunohistochemistry of α‐SMA and Ki‐67 in cross‐sections of the adductor muscles collected from NFAT5 knockdown rats treated with MCP‐1 delivery 21 d after FAL. D, Quantification of images from (C), collateral artery diameter and lumen areas were assayed by α‐SMA staining, and proliferating cells were measured by Ki‐67 staining. Scale bar = 50 μm. **P < .01 vs + PBS; N = 6 rats in each group

As we have proved that NFAT5 knockdown in ECs may result in the reduction of monocytes chemotaxis through MCP‐1, which in turn leading a decreased infiltration of macrophages. Therefore, we investigated whether MCP‐1 delivery could reverse the impairment of collateral circulations growth result from NFAT5 knockdown. As shown in Figure 6A, laser speckle contrast imaging demonstrated that MCP‐1 delivery into rats hindlimb muscles could markedly restore the blood perfusion of hindlimb that was inhibited by NFAT5 knockdown at day 3, 7, 14 and 21 after surgery (day 3, 33.3 ± 6.1% vs 48.5 ± 4.6%, *P* < .01; day 7, 47.5 ± 5.5% vs 69.8 ± 6.1%, *P* < .01; day 14, 60.5 ± 4.0% vs 76.2 ± 5.2%, *P* < .01; day 21, 69.3 ± 6.1% vs 85.9 ± 6.6%, *P* < .01). Similarly, corresponding angiographies of MCP‐1‐treated rat hindlimbs illustrated good collaterals growth when NFAT5 knockdown (Figure S3). Besides, immunohistochemistry of CD68 indicated that MCP‐1 obviously promoted macrophages to infiltrate into the adductors when NFAT5 knockdown (8 ± 3 vs 20 ± 4, *P* < .01, Figure 6B). Next, to explore whether MCP‐1 reverse the inhibitory effect of NFAT5 knockdown on collateral vessels growth, we examined the adductors 21 days after FAL. Consequently, the induction of arteriogenesis in the adductor was better in MCP‐1‐treated rats compared with control rats in condition of NFAT5 knockdown, which was reflected by significant increases in artery diameter (33 ± 4 vs 46 ± 7 μm, *P* < .01), artery area (566 ± 115 vs 1312 ± 182 μm^2^, *P* < .01), Ki‐67 expression (12 ± 3 vs 25 ± 5, *P* < .01) and proliferative index (0.25 ± 0.05 vs 0.44 ± 0.07; *P* < .01, Figure 6C,D).

### 3.6 Angiogenesis is also impaired in rats lacking NFAT5

We also investigated whether NFAT5 is required for angiogenesis during collateral circulations growth. Our results showed that injection with Ad‐shNFAT5 in gastrocnemius significantly impaired the blood flow recovery in the hindlimbs (day 3, 53.3 ± 6.1% vs 41.9 ± 7.6%, *P* < .01; day 7, 72.6 ± 5.5% vs 52.8 ± 4.3%, *P* < .01; day 14, 86.2 ± 4.0% vs 72.7 ± 5.3%, *P* < .01; day 21, 90.1 ± 7.3% vs 77.6 ± 4.2%, *P* < .01; Figure S4A). Meanwhile, macrophages infiltration at day 7 (19.7 ± 4.5 vs 5.3 ± 3.4, *P* < .01; Figure S4B) and the number of capillaries at day 21 (58.2 ± 11.3 vs 23.7 ± 4.4, *P* < .01; Figure S4B) in Ad‐shNFAT5‐treated rats were also weakened when compared with Ad‐null‐treated rats.

## 4 DISCUSSION

The primary findings of the present study are as follows: (a) genetical silence of NFAT5 attenuates arteriogenesis and angiogenesis in rat hindlimb ischaemia model; (b) NFAT5 can regulate monocyte recruitment by regulating the expression of MCP‐1 in ECs both in vivo and in vitro; (c) MCP‐1 supplementation can reverse the inhibitory effect of NFAT5 knockdown on monocyte recruitment and arteriogenesis. Collectively, these findings mainly suggest that NFAT5 promotes arteriogenesis via MCP‐1‐dependent monocyte recruitment.

Hindlimb ischaemia model showed that NFAT5 increased since the 12th hour after FAL, reached the highest level at the 3rd day, began to decrease from the 7th day and returned to normal level at the 14th day. After NFAT5 was knocked down, the blood flow recovery of the lower limbs was inhibited. The diameter and covering area of collateral arteries were decreased, as well as the number of proliferative cells, implying that arteriogenesis was inhibited by NFAT5 knockdown after FAL. Therefore, these results indicated that NFAT5 can promote collateral artery formation. It was evidenced that the 3rd day when NFAT5 increased maximally is also the time when maximal monocyte recruitment occurred.^23^, ^29^ Compelling evidence has indicated an essential role for monocytes in arteriogenesis.^30^, ^31^ Specifically, in the rabbit and mouse hindlimb ischaemia models, pharmacological monocyte depletion led to impaired arteriogenesis which can be rescued by restoring monocyte blood count through the injection of exogenous cells.^32^ In keeping with these results, the magnitude of their infiltration at the site of collateral arteries positively correlates with arteriogenesis development.^33^, ^34^ Our experiments showed that after NFAT5 knockdown, the formation of collateral arteries was inhibited, and the aggregation of macrophages around collateral arteries was significantly inhibited on the 3rd day after ligation as was shown with CD68 immunochemistry assays. At the same time, immunofluorescence assays showed that NFAT5 was mainly expressed in the endothelium of collateral artery. Therefore, we speculated that endothelial NFAT5 might regulate arteriogenesis by regulating monocyte recruitment.

Next, we explored the mechanism of NFAT5 regulating monocyte recruitment. According to previous study, ICAM‐1, VCAM‐1 and MCP‐1 are the three predominant up‐regulated molecules that induce monocyte recruitment during arteriogenesis.^6^, ^35^ Our results showed that NFAT5 knockdown did not affect ICAM‐1 and VCAM‐1 mRNA expression, but both mRNA and protein expressions of MCP‐1 were notably decreased, and MCP‐1 around collateral arteries was also decreased. Apart from the importance of monocytes for arteriogenesis, it is apparent that MCP‐1 plays a pivotal role for monocyte recruitment during arteriogenesis. In different animal models of FAL, infusion of MCP‐1 led to an increased number of macrophages surrounding growing collaterals and improved collateral flow.^36^, ^37^, ^38^ By contrast, mice genetically deficient for MCP‐1 or its isogenous receptor CCR2 displayed reduced monocyte infiltration and decreased arteriogenesis following artery occlusion.^39^, ^40^ Moreover, clinical data also demonstrated that in the early stage of acute myocardial infarction, patients with high plasma level of MCP‐1 developed better collateral vessels than those with low level.^41^ From the evidence above, we have reason to believe that NFAT5 affects monocyte recruitment by regulating MCP‐1 expression, which in turn to affect arteriogenesis.

In order to further verify the molecular mechanism of NFAT5 affecting arteriogenesis, we carried out experiments in vitro. The expression of NFAT5 and nuclear transfer were increased in HUVECs when stimulated by IL‐1β. NFAT5 knockdown inhibited IL‐1β induced MCP‐1 secretion and the chemotaxis of THP‐1 cells. Thus, NFAT5 could regulate monocyte recruitment via regulating the expression of MCP‐1. Besides, IL‐1β was also suggested to promote ischaemia‐induced neovascularization via mobilizing CD34^−^/B220^−^CD3^–^Flk‐1^+^ endothelial precursor cells (EPCs), however, whether NFAT5 involves in this process as well as affect EPCs needed further study.^42^

It should be noted that in the early stage of arteriogenesis, MCP‐1 is mainly secreted by the activated endothelium induced by hemodynamic forces,^43^ which is followed by accumulation of a large number of macrophages that release a variety of inflammatory cytokines (such as IL‐1β, TNF‐α). These cytokines potently induce MCP‐1 expression in arteries. Our study showed that the expression of NFAT5 peaked at the 3rd day when it was also found, by immunofluorescence and immunohistochemistry, to be mainly expressed in the endothelial cells of collateral arteries surrounded by numerous macrophages and when the ECs are stimulated strongly by inflammatory cytokines. Hence, it is reasonable for using ECs stimulated by inflammatory cytokines to explore the cellular regulatory role of NFAT5 in the process of arteriogenesis. Moreover, animal studies showed that NFAT5 increased as early as 12 hours after FAL when the ECs are mainly stimulated by mechanical forces. Thus, exploring the effect of mechanical stimulation on NFAT5 in ECs and its mechanism is still necessary, which is also the limitation of this study and worth further studies.

In addition, we explored the possible upstream pathway that might affect the expression of NFAT5 and MCP‐1. Previous studies showed that endothelial ERK1/2 pathway is critical for arteriogenesis,^44^, ^45^ and ERK1/2 pathway is also involved in the regulation of MCP‐1 expression via NFAT5 in NRK52E cells.^46^ Besides, activation of ERK1/2 pathway is required for IL‐1β induced MCP‐1 expression in various cells.^47^, ^48^, ^49^ Therefore, we have been suggested that ERK1/2 pathway might be the upstream pathway in this study. Accordingly, our experiments in vitro showed that the expression of NFAT5 and MCP‐1, as well as the chemotaxis of THP‐1 cells were attenuated after ERK1/2 pathway was inhibited by U0126. To summarize, ERK1/2 pathway may be involved in the regulation of MCP‐1 expression via NFAT5 and thus promote monocyte recruitment during arteriogenesis.

In order to further confirm the upstream and downstream relationship between NFAT5 and MCP‐1 in the formation of collateral arteries, we performed an exogenous MCP‐1 supplement to NFAT5 knockdown rats with FAL. We found that the MCP‐1 supplement group showed increased diameter, and covering area of collateral arteries, as well as greater number of proliferating cells, enhanced macrophages infiltration and better blood flow recovery. Therefore, the inhibitory effect on arteriogenesis induced by NFAT5 knockdown could be reversed by MCP‐1 supplement, demonstrating that NFAT5 plays a role in promoting arteriogenesis through MCP‐1 pathway.

Previous study also showed that NFAT5 can promote angiogenesis both in vitro and in vivo.^26^ Our result showed that when injection of Ad‐NFAT5 into gastrocnemius, macrophage infiltration and density of capillaries in gastrocnemius as well as blood flow of lower limbs were also inhibited. Therefore, NFAT5 is also required for angiogenesis in hindlimb ischaemia rats dependent on monocyte recruitment.

In summary, our results demonstrated that NFAT5 can promote arteriogenesis via MCP‐1‐dependent monocyte recruitment, suggesting that NFAT5 may be an endogenous regulator of arteriogenesis. Therefore, targeting NFAT5 may be considered as a novel strategy in the treatment of ischaemic diseases.

## CONFLICTS OF INTEREST

The authors declare no conflicts of interest.

## AUTHOR CONTRIBUTION

GGZ and CCL conceived the project, funded this study and revised the manuscript. XCL designed and performed most experiments, performed statistical analysis and wrote the manuscript. MP, LPZ, QS and ZSZ partially performed some experiments. We also thank Professor Wei‐Jun Cai for his kind guidance of our animal experiments.

## Data Availability

The data that support the findings of this study are available from the corresponding author upon reasonable request.

## References

[1] Li R , Lan B , Zhu T , et al. Preventing graft restenosis after coronary artery bypass grafting with tissue‐type plasminogen activator. Eur J Med Res. 2017;22:18. 28606123 10.1186/s40001-017-0259-8 PMC5469182

[2] Marino BC , Nascimento GA , Rabelo W , et al. Clinical coronary in‐stent restenosis follow‐up after treatment and analyses of clinical outcomes. Arq Bras Cardiol. 2015;104:375‐386. 25651344 10.5935/abc.20140216 PMC4495452

[3] Elias J , Hoebers LPC , van Dongen IM , et al. Impact of collateral circulation on survival in ST‐segment elevation myocardial infarction patients undergoing primary percutaneous coronary intervention with a concomitant chronic total occlusion. JACC Cardiovasc Interv. 2017;10:906‐914. 28473112 10.1016/j.jcin.2017.01.026

[4] Möbius‐Winkler S , Uhlemann M , Adams V , et al. Coronary collateral growth induced by physical exercise: results of the impact of intensive exercise training on coronary collateral circulation in patients with stable coronary artery disease (EXCITE) trial. Circulation. 2016;133:1438‐1448. 26979085 10.1161/CIRCULATIONAHA.115.016442

[5] Zhang H , Faber JE . De‐novo collateral formation following acute myocardial infarction: Dependence on CCR2⁺ bone marrow cells. J Mol Cell Cardiol. 2015;87:4‐16. 26254180 10.1016/j.yjmcc.2015.07.020 PMC4637183

[6] Simons M , Eichmann A . Molecular controls of arterial morphogenesis. Circ Res. 2015;116:1712‐1724. 25953926 10.1161/CIRCRESAHA.116.302953 PMC4509635

[7] Cai W , Schaper W . Mechanisms of arteriogenesis. Acta Biochim Biophys Sin (Shanghai). 2008;40:681‐692. 18685784

[8] Wu S , Wu X , Zhu W , et al. Immunohistochemical study of the growth factors, aFGF, bFGF, PDGF‐AB, VEGF‐A and its receptor (Flk‐1) during arteriogenesis. Mol Cell Biochem. 2010;343:223‐229. 20559689 10.1007/s11010-010-0517-3

[9] Shireman PK . The chemokine system in arteriogenesis and hind limb ischemia. J Vasc Surg. 2007;45(6):A48‐A56. 17544024 10.1016/j.jvs.2007.02.030 PMC2680944

[10] Baeyens N , Bandyopadhyay C , Coon BG , et al. Endothelial fluid shear stress sensing in vascular health and disease. J Clin Invest. 2016;126:821‐828. 26928035 10.1172/JCI83083 PMC4767335

[11] Zimarino M , D'Andreamatteo M , Waksman R , et al. The dynamics of the coronary collateral circulation. Nat Rev Cardiol. 2014;11:191‐197. 24395049 10.1038/nrcardio.2013.207

[12] Lee N , Kim D , Kim WU . Role of NFAT5 in the Immune System and Pathogenesis of Autoimmune Diseases. Front Immunol. 2019;10:270. 30873159 10.3389/fimmu.2019.00270 PMC6401628

[13] Aramburu J , Drews‐Elger K , Estrada‐Gelonch A , et al. Regulation of the hypertonic stress response and other cellular functions by the Rel‐like transcription factor NFAT5. Biochem Pharmacol. 2006;72:1597‐1604. 16904650 10.1016/j.bcp.2006.07.002

[14] Kim DH , Kim KS , Ramakrishna S . NFAT5 promotes in vivo development of murine melanoma metastasis. Biochem Biophys Res Commun. 2018;505:748‐754. 30293684 10.1016/j.bbrc.2018.09.171

[15] Choi S , You S , Kim D , et al. Transcription factor NFAT5 promotes macrophage survival in rheumatoid arthritis. J Clin Invest. 2017;127:954‐969. 28192374 10.1172/JCI87880 PMC5330733

[16] Cao W , Zhang D , Li Q , et al. Biomechanical Stretch Induces Inflammation, Proliferation, and Migration by Activating NFAT5 in Arterial Smooth Muscle Cells. Inflammation. 2017;40:2129‐2136. 28840417 10.1007/s10753-017-0653-y

[17] Scherer C , Pfisterer L , Wagner AH , et al. Arterial wall stress controls NFAT5 activity in vascular smooth muscle cells. J Am Heart Assoc. 2014;3:e000626. 24614757 10.1161/JAHA.113.000626 PMC4187483

[18] Hernández GL , Volpert OV , Iñiguez MA , et al. Selective inhibition of vascular endothelial growth factor‐mediated angiogenesis by cyclosporin A: roles of the nuclear factor of activated T cells and cyclooxygenase 2. J Exp Med. 2001;193:607‐620. 11238591 10.1084/jem.193.5.607 PMC2193389

[19] Hödebeck M , Scherer C , Wagner AH , et al. TonEBP/NFAT5 regulates ACTBL2 expression in biomechanically activated vascular smooth muscle cells. Front Physiol. 2014;5:467. 25520667 10.3389/fphys.2014.00467 PMC4253659

[20] Berry MR , Mathews RJ , Ferdinand JR , et al. Renal sodium gradient orchestrates a dynamic antibacterial defense zone. Cell. 2017;170(860–874):e19. 10.1016/j.cell.2017.07.022 28803730

[21] Troidl K , Rüding I , Cai WJ , et al. Actin‐binding rho activating protein (Abra) is essential for fluid shear stress‐induced arteriogenesis. Arterioscler Thromb Vasc Biol. 2009;29:2093‐2101. 19778941 10.1161/ATVBAHA.109.195305

[22] Luo MY , Yang BL , Ye F , et al. Collateral vessel growth induced by femoral artery ligature is impaired by denervation. Mol Cell Biochem. 2011;354:219‐229. 21509579 10.1007/s11010-011-0821-6

[23] Khmelewski E , Becker A , Meinertz T , Ito WD . Tissue resident cells play a dominant role in arteriogenesis and concomitant macrophage accumulation. Circ Res. 2004;95:E56‐E64. 15331452 10.1161/01.RES.0000143013.04985.E7

[24] Zhu LP , Zhou JP , Zhang JX , et al. MiR‐15b‐5p regulates collateral artery formation by targeting AKT3 (Protein Kinase B‐3). Arterioscler Thromb Vasc Biol. 2017;37:957‐968. 28254819 10.1161/ATVBAHA.116.308905

[25] Sweet DT , Chen Z , Givens CS , et al. Endothelial Shc regulates arteriogenesis through dual control of arterial specification and inflammation via the notch and nuclear factor‐κ‐light‐chain‐enhancer of activated B‐cell pathways. Circ Res. 2013;113:32‐39. 23661718 10.1161/CIRCRESAHA.113.301407 PMC3918667

[26] Yoon HJ , You S , Yoo SA , et al. NF‐AT5 is a critical regulator of inflammatory arthritis. Arthritis Rheum. 2011;63:1843‐1852. 21717420 10.1002/art.30229 PMC3084342

[27] Makó V , Czúcz J , Weiszhár Z , et al. Proinflammatory activation pattern of human umbilical vein endothelial cells induced by IL‐1β, TNF‐α, and LPS. Cytometry A. 2010;77:962‐970. 21290470 10.1002/cyto.a.20952

[28] Yoshimura H , Nakahama K , Safronova O , et al. Transforming growth factor‐beta stimulates IL‐1beta‐induced monocyte chemoattractant protein‐1 expression in human synovial cells via the ERK/AP‐1 pathway. Inflamm Res. 2006;55:543‐549. 17039283 10.1007/s00011-006-5144-9

[29] Ito WD , Lund N , Zhang Z , et al. Activation of cell surface Bound 20S proteasome inhibits vascular cell growth and arteriogenesis. Biomed Res Int. 2015;2015:719316. 26146628 10.1155/2015/719316 PMC4471257

[30] Bruce AC , Kelly‐Goss MR , Heuslein JL , et al. Monocytes are recruited from venules during arteriogenesis in the murine spinotrapezius ligation model. Arterioscler Thromb Vasc Biol. 2014;34:2012‐2022. 24969773 10.1161/ATVBAHA.114.303399 PMC4373588

[31] Krishnasamy K , Limbourg A , Kapanadze T , et al. Blood vessel control of macrophage maturation promotes arteriogenesis in ischemia. Nat Commun. 2017;8:952. 29038527 10.1038/s41467-017-00953-2 PMC5643305

[32] Fung E , Helisch A Macrophages in collateral arteriogenesis. Front Physiol. 2012;3:353. 23055975 10.3389/fphys.2012.00353 PMC3457069

[33] Hakimzadeh N , Verberne HJ , Siebes M , Piek JJ . The future of collateral artery research. Curr Cardiol Rev. 2014;10:73‐86. 23638829 10.2174/1573403X113099990001 PMC3968596

[34] Pipp F , Heil M , Issbrücker K , et al. VEGFR‐1‐selective VEGF homologue PlGF is arteriogenic: evidence for a monocyte‐mediated mechanism. Circ Res. 2003;92:378‐385. 12600898 10.1161/01.RES.0000057997.77714.72

[35] Núñez‐Gómez E , Pericacho M , Ollauri‐Ibáñez C , et al. The role of endoglin in post‐ischemic revascularization. Angiogenesis. 2017;20:1‐24. 27943030 10.1007/s10456-016-9535-4

[36] Ogle ME , Segar CE , Sridhar S , Botchwey EA . Monocytes and macrophages in tissue repair: Implications for immunoregenerative biomaterial design. Exp Biol Med (Maywood). 2016;241:1084‐1097. 27229903 10.1177/1535370216650293 PMC4898192

[37] Hollander MR , Horrevoets AJ , van Royen N . Cellular and pharmacological targets to induce coronary arteriogenesis. Curr Cardiol Rev. 2014;10:29‐37. 23638831 10.2174/1573403X113099990003 PMC3968592

[38] Sapharikas E , Lokajczyk A , Fischer AM , Boisson‐Vidal C . Fucoidan Stimulates Monocyte Migration via ERK/p38 Signaling Pathways and MMP9 Secretion. Mar Drugs. 2015;13:4156‐4170. 26133555 10.3390/md13074156 PMC4515609

[39] Voskuil M , Hoefer IE , van Royen N , et al. Abnormal monocyte recruitment and collateral artery formation in monocyte chemoattractant protein‐1 deficient mice. Vasc Med. 2004;9:287‐292. 15678621 10.1191/1358863x04vm571oa

[40] Heil M , Ziegelhoeffer T , Wagner S , et al. Collateral artery growth (arteriogenesis) after experimental arterial occlusion is impaired in mice lacking CC‐chemokine receptor‐2. Circ Res. 2004;94:671‐677. 14963007 10.1161/01.RES.0000122041.73808.B5

[41] Sahinarslan A , Kocaman SA , Topal S , et al. Relation between serum monocyte chemoattractant protein‐1 and coronary collateral development. Coron Artery Dis. 2010;21:455‐459. 20859200 10.1097/MCA.0b013e32833fd29b

[42] Amano K , Okigaki M , Adachi Y , et al. Mechanism for IL‐1 beta‐mediated neovascularization unmasked by IL‐1 beta knock‐out mice. J Mol Cell Cardiol. 2004;36:469‐480. 15081307 10.1016/j.yjmcc.2004.01.006

[43] Limbourg A , von Felden J , Jagavelu K , et al. MAP‐kinase activated protein kinase 2 links endothelial activation and monocyte/macrophage recruitment in arteriogenesis. PLoS ONE. 2015;10:e0138542. 26431421 10.1371/journal.pone.0138542 PMC4592267

[44] Kofler NM , Simons M . Angiogenesis versus arteriogenesis: neuropilin 1 modulation of VEGF signaling. F1000Prime Rep. 2015;7:26. 25926977 10.12703/P7-26 PMC4371373

[45] Ren B . Protein kinase D1 signaling in angiogenic gene expression and VEGF‐mediated angiogenesis. Front Cell Dev Biol. 2016;4:37. 27200349 10.3389/fcell.2016.00037 PMC4854877

[46] Kojima R , Taniguchi H , Tsuzuki A , et al. Hypertonicity‐induced expression of monocyte chemoattractant protein‐1 through a novel cis‐acting element and MAPK signaling pathways. J Immunol. 2010;184:5253‐5262. 20368270 10.4049/jimmunol.0901298

[47] Chang MC , Tsai YL , Chang HH , et al. IL‐1β‐induced MCP‐1 expression and secretion of human dental pulp cells is related to TAK1, MEK/ERK, and PI3K/Akt signaling pathways. Arch Oral Biol. 2016;61:16‐22. 26492523 10.1016/j.archoralbio.2015.10.008

[48] Bian ZM , Elner SG , Yoshida A , et al. Activation of p38, ERK1/2 and NIK pathways is required for IL‐1beta and TNF‐alpha‐induced chemokine expression in human retinal pigment epithelial cells. Exp Eye Res. 2001;73:111‐121. 11428868 10.1006/exer.2001.1019

[49] Chen MC , Proost P , Gysemans C , et al. Monocyte chemoattractant protein‐1 is expressed in pancreatic islets from prediabetic NOD mice and in interleukin‐1 beta‐exposed human and rat islet cells. Diabetologia. 2001;44:325‐332. 11317664 10.1007/s001250051622

