# Supplementary figures and images for: NFAT5 promotes arteriogenesis via MCP‐1‐dependent monocyte recruitment
Source: J Cell Mol Med. 2019 Dec 28;24(2):2052–63. doi: 10.1111/jcmm.14904 (PMC6991654; doi:10.1111/jcmm.14904)

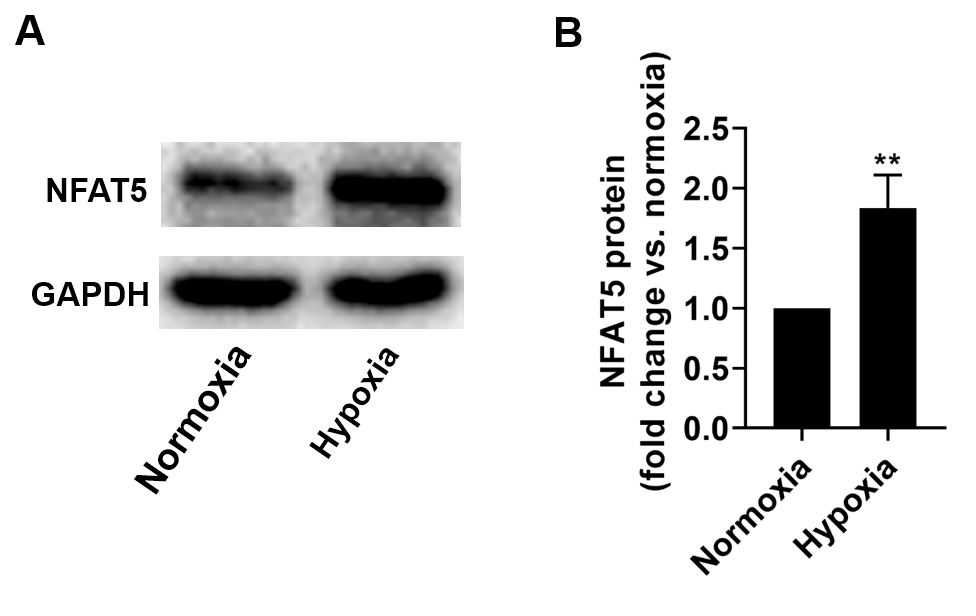

Supplement: Supplementary file 1 [file JCMM-24-2052-s001.tif]

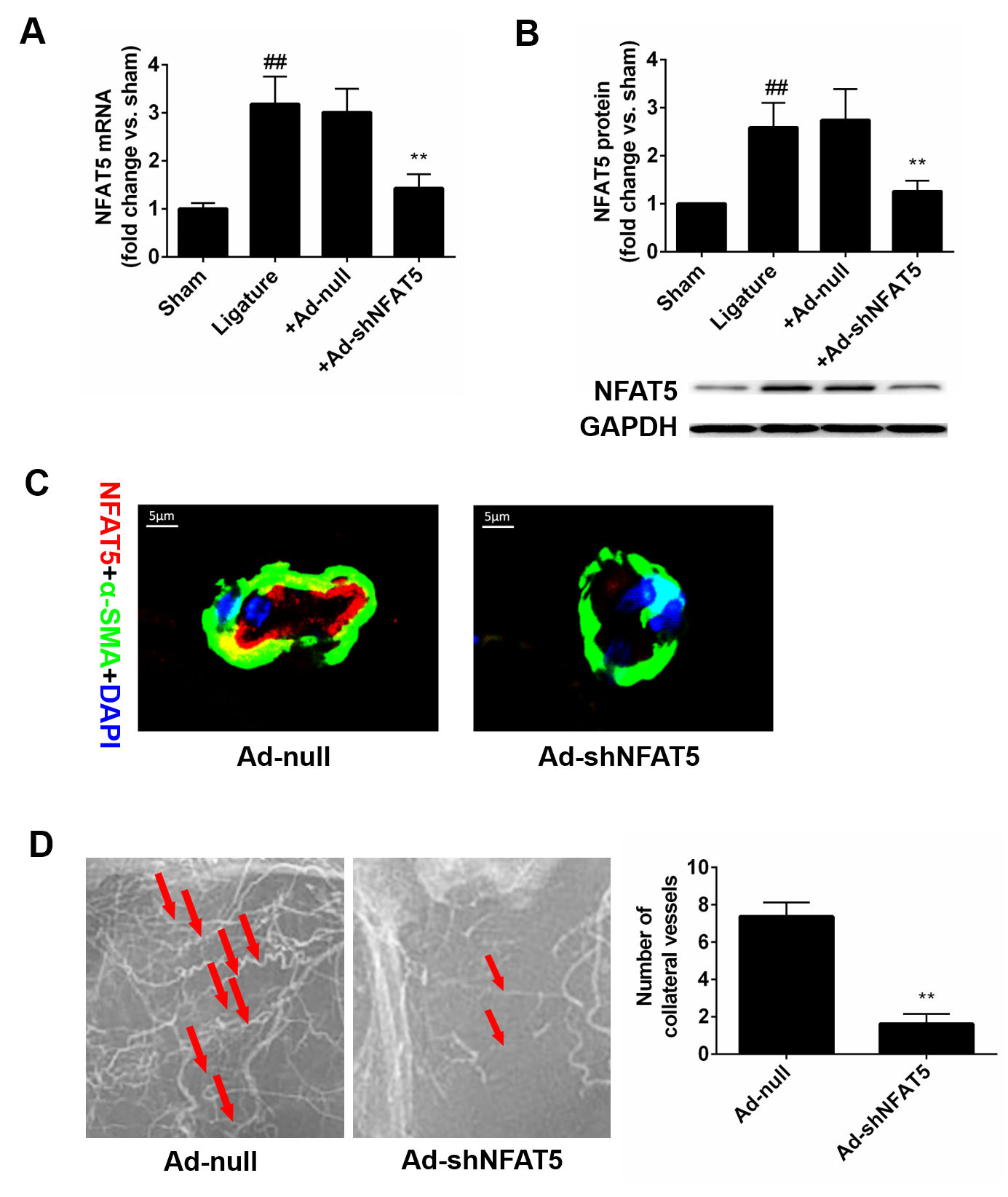

Supplement: Supplementary file 2 [file JCMM-24-2052-s002.tif]

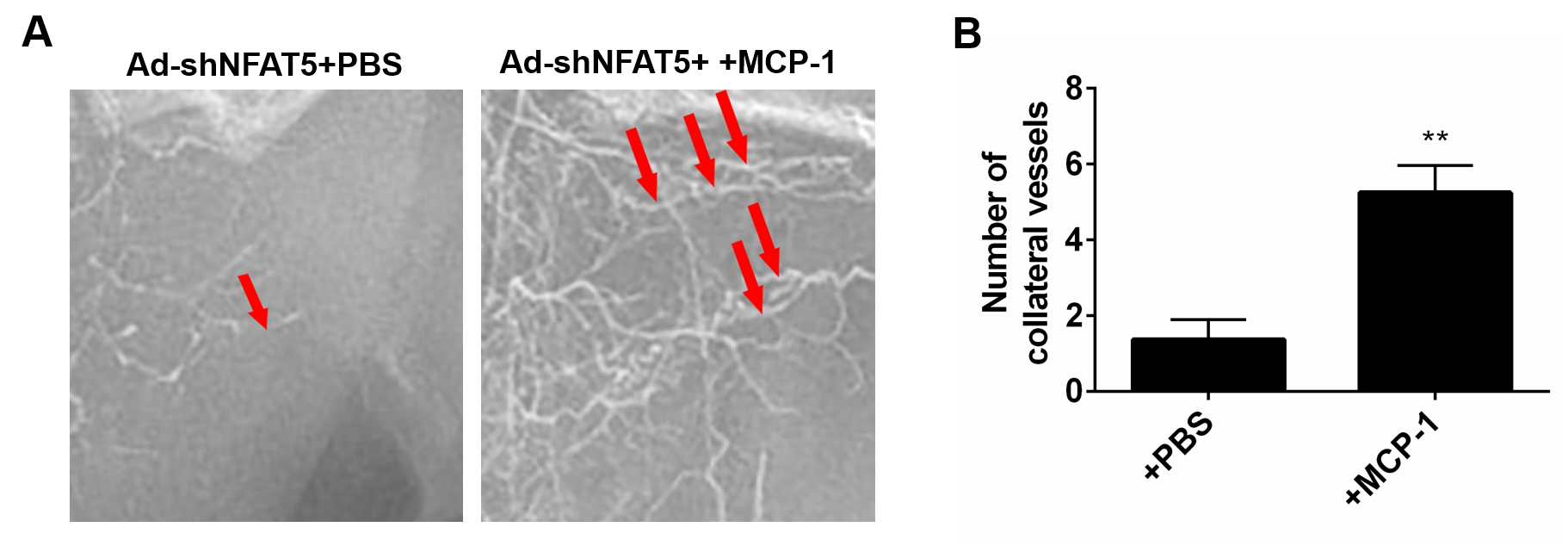

Supplement: Supplementary file 3 [file JCMM-24-2052-s003.tif]

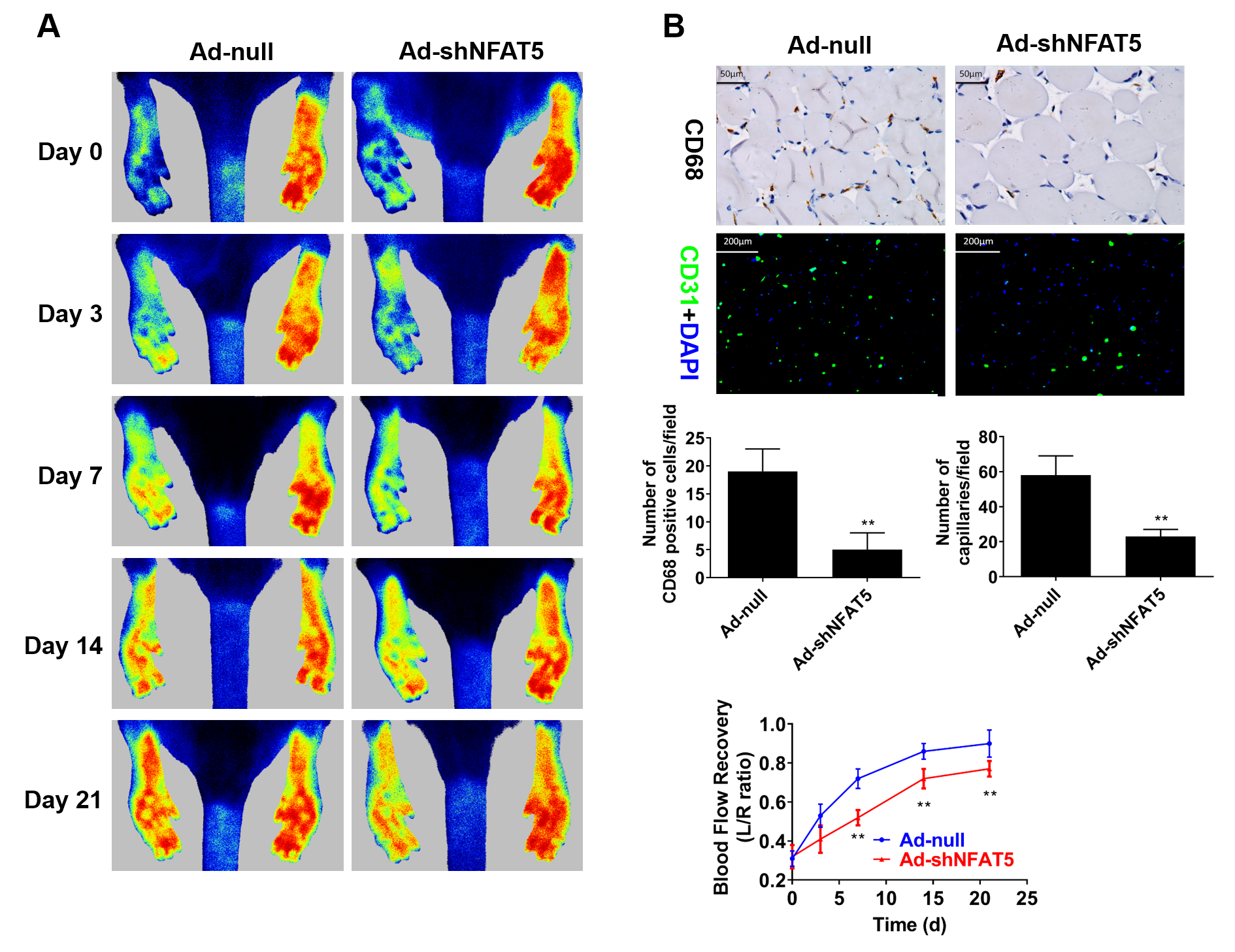

Supplement: Supplementary file 4 [file JCMM-24-2052-s004.tif]
