# Supplementary material for: NFAT5 promotes arteriogenesis via MCP‐1‐dependent monocyte recruitment
Source: J Cell Mol Med. 2019 Dec 28;24(2):2052–63. doi: 10.1111/jcmm.14904 (PMC6991654; doi:10.1111/jcmm.14904)
Supplement: Supplementary file 5 [file JCMM-24-2052-s005.docx]

**Table S1** The primer sequences used for real-time PCR and their expected product size

| Gene names | species | Primer sequences (5´ to 3´) | Product size (bp) |
| --- | --- | --- | --- |
| NFAT5 | Rat | F: ATGTTGTGACCAATGCTGGA | 204 |
|  |  | R: TGATCAGGGCATTAGGAAGG |  |
| ICAM-1 | Rat | F: AGCTCCGGACTTTCGATCTT | 120 |
|  |  | R: CTTCAGAGGCAGGAAACAGG |  |
| VCAM-1 | Rat | F: GTCAGCGAAGGAAACTGGAG | 121 |
|  |  | R: ACCGTGCAGTTGACAGTGAC |  |
| MCP-1 | Rat | F: TAGCATCCACGTGCTGTCTC | 122 |
|  |  | R: TGCTGCTGGTGATTCTCTTG |  |
| GAPDH | Rat | F: GAAGGGCTCATGACCACAGT | 117 |
|  |  | R: GGATGCAGGGATGATGTTCT |  |
| NFAT5 | Human | F: CAACAAGCTGCTTTCCAACA | 161 |
|  |  | R: ATGGTTCCTTGTTGGCTTTG |  |
| MCP-1 | Human | F: CCCCAGTCACCTGCTGTTAT | 171 |
|  |  | R: TGGAATCCTGAACCCACTTC |  |
| GAPDH | Human | F: CTGCACCACCAACTGCTTAG | 282 |
|  |  | R: AGGTCCACCACTGACACGTT |  |

F indicates forward primer; R indicates reverse primer.
